# Characterization, in Vivo and in Vitro Evaluation of Solid Dispersion of Curcumin Containing d-α-Tocopheryl Polyethylene Glycol 1000 Succinate and Mannitol

**DOI:** 10.3390/molecules21101386

**Published:** 2016-10-17

**Authors:** Im-Sook Song, Jin-Sun Cha, Min-Koo Choi

**Affiliations:** 1 College of Pharmacy and Research Institute of Pharmaceutical Sciences, Kyungpook National University, Daegu 41566, Korea; isssong@knu.ac.kr; 2 College of Pharmacy, Dankook University, Cheon-an 31116, Korea; tbuma@dreamwiz.com

**Keywords:** curcumin, TPGS, solid dispersion, dissolution, oral bioavailability

## Abstract

The aim of this study was to prepare a solid dispersion formulation of curcumin to enhance its solubility, dissolution rate, and oral bioavailability. The formulation was prepared with d-α-tocopheryl polyethylene glycol 1000 succinate (TPGS) and mannitol using solvent evaporation and freeze-drying methods, which yielded a solid dispersion composed of curcumin, TPGS, and mannitol at a ratio of 1:10:15 (*w*/*w*/*w*). The solubility and dissolution rate of the curcumin solid dispersion markedly improved compared with those of curcumin powder and a physical mixture of curcumin, TPGS, and mannitol. About 90% of the curcumin was released from the solid dispersion formulation within 10 min. After administering the formulation orally to rats, higher plasma concentrations of curcumin were observed, with increases in the maximum plasma concentration (C_max_) and area under the plasma concentration-time curve (AUC) of 86- and 65-fold, respectively, compared with those of curcumin powder. The solid dispersion formulation effectively increased intestinal permeability and inhibited P-gp function. These effects increased the anti-proliferative effect of curcumin in MDA-MB-231 breast cancer cells. Moreover, 2 h incubation with curcumin powder, solid dispersion formulation, and its physical mixture resulted in differential cytotoxic effect of paclitaxel in P-gp overexpressed LLC-PK1-P-gp and MDA-MB-231 cells through the inhibition of P-gp-mediated paclitaxel efflux. In conclusion, compared with curcumin, a solid dispersion formulation of curcumin with TPGS and mannitol could be a promising option for enhancing the oral bioavailability and efficacy of curcumin through increased solubility, dissolution rate, cell permeability, and P-gp modulation.

## 1. Introduction

Curcumin, 1,7-bis(4-hydroxy-3-methoxyphenyl)-1,6-heptadiene-3,5-dione, is extracted from the rhizome of *Curcuma longa *L. It has long been used in Asian countries as a spice and herbal medicine [1]. Curcumin has anti-bacterial, anti-coagulant, anti-oxidant, and anti-inflammatory properties [2]. Additionally, curcumin has been found to be efficacious against many types of cancer. [3] In particular, curcumin has been reported to inhibit breast cancer cell motility [4]. The underlying mechanisms were intensively investigated. Curcumin decreased the proliferation of MDA-MB-435 breast cancer cells by down-regulating mitogen-activated protein kinase (MAPK) pathway-mediated enhancement of the zeste homolog 2 (EZH2) gene [5]. It also inhibited the proliferation of MDA-MB-231 breast cancer cells either by up-regulating p21 expression or the ratio of Bax to Bcl-2 [6].

However, the oral bioavailability of curcumin is very low, which can be a barrier to achieving therapeutic effects with this drug. The limiting factors for the oral absorption of curcumin include its poor aqueous solubility and intestinal permeability, and extensive systemic metabolism [7,8,9,10]. Curcumin was found to be practically insoluble in water, with a maximum solubility as low as 11 ng/mL in aqueous buffer (pH 5.0) [11]. Recently, the low permeability of curcumin in Caco-2 cells and its enterocytes-based metabolism by CYP450 3A4 have been reported [8,12]. Moreover, the involvement of P-glycoprotein (P-gp) in curcumin transport has been reported [13,14]. Thus, improving the solubility and intestinal permeability are important in enhancing the bioavailability of curcumin. Since P-gp has been reported to be a barrier to the anti-cancer effects of various drugs, the modulation of P-gp substrate specificity of curcumin would improve its intestinal absorption and anti-cancer efficacy.

d-α-tocopheryl polyethylene glycol 1000 succinate (TPGS) is a non-ionic surfactant with a critical micelle concentration (CMC) of 0.02% (*w*/*w*) and a hydrophile-lipophile balance (HLB) value of 13.2 [15]. TPGS is widely used as a solubilizing agent, an emulsifier, a permeation enhancer, and a stabilizer in drug formulations. Specifically, TPGS inhibits P-gp function, thereby increasing the intestinal absorption and cellular accumulation of its substrate drugs [16,17,18]. Moreover, US FDA has approved TPGS as a safe pharmaceutical excipient for use in drug formulation [19].

The solid dispersion system is one of the widely used strategies for enhancing the bioavailability of poorly soluble drugs [20]. In this system, the drug can exist as molecular dispersion and/or amorphous form within hydrophilic carriers. The resulting improved solubility and dissolution rate are achieved through particle size reduction and surface area enhancement of the drug [21]. Additionally, the use of hydrophilic carriers increases the wettability of hydrophobic drugs [20,22].

Therefore, in the present study, we developed an oral solid dispersion formulation of curcumin using TPGS as a surfactant and a P-gp inhibitor, with mannitol as a hydrophilic carrier. We then evaluated the physicochemical characteristics, dissolution behavior, oral bioavailability in rats, as well as the efficacy and related mechanisms for the curcumin-loaded solid dispersion formulation.

## 2. Results

### 2.1. Preparation of Curcumin-Loaded Solid Dispersion

**Figure 1:**
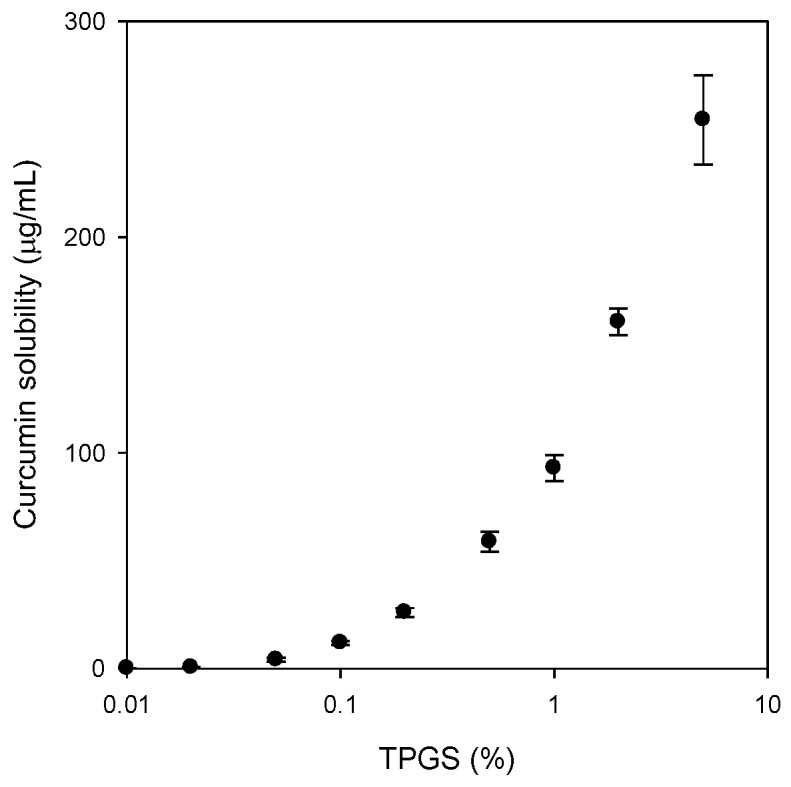
Solubility of curcumin was determined with increasing amount of TPGS. Each bar represents the mean ± S.D. of three independent experiments.

To confirm the solubility enhancement by TPGS, curcumin solubility was measured in the presence of various concentrations of TPGS. As shown in Figure 1, the aqueous solubility of curcumin was determined as 6.6 ng/mL, and this increased dramatically in a TPGS concentration-dependent manner, especially over the critical micelle concentration (CMC) of TPGS (0.05% in this study). The solubility enhancement of curcumin is affected by TPGS micelle formation [15].

**Figure 2:**
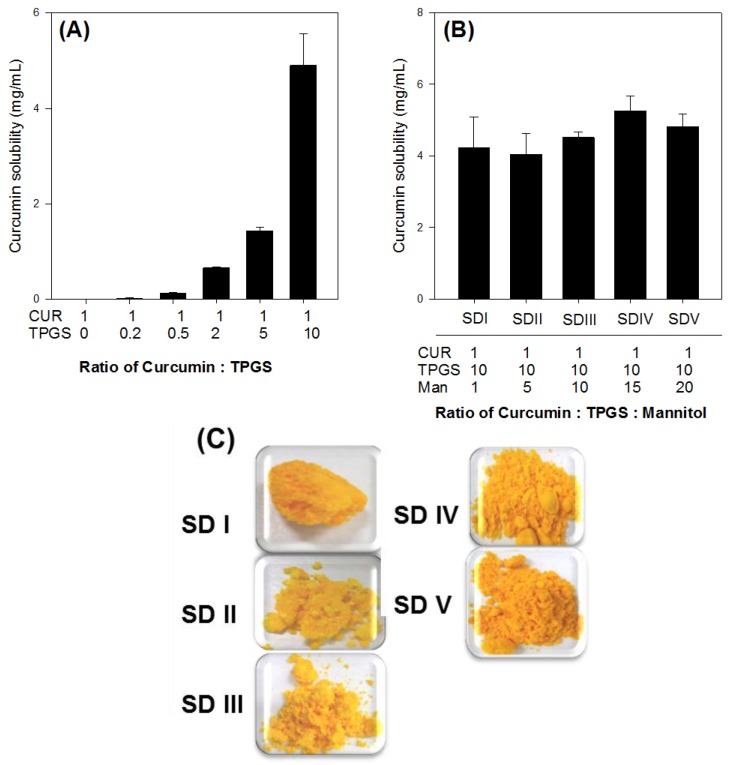
Solubility of curcumin in a solid dispersion (SD I–V) formulation. Each bar represents the mean ± S.D. of three independent experiments. (**A**) Solubility of curcumin was determined with increasing amount of TPGS; (**B**) Solubility of curcumin was determined with different ratio of curcumin: TPGS: mannitol; (**C**) Physical status of solid dispersion with different ratio of curcumin:TPGS:mannitol.

To determine the optimal ratio of curcumin to TPGS for the curcumin-loaded solid dispersion formulation, we measured the solubility of curcumin in various curcumin-TPGS composition mixtures. The solubility of curcumin in a dried waxy mass, prepared with TPGS by the solvent evaporation method, increased in a TPGS-dependent manner (Figure 2A). Based on this result, the optimal ratio of curcumin to TPGS was determined to be 1:10 (*w*/*w*).

To prepare curcumin-loaded solid dispersion in a solid powder form, we co-formulated with mannitol as a hydrophilic carrier. The optimal composition was selected based on curcumin solubility and physical properties of the formulations. The amount of mannitol was increased while maintaining a fixed ratio of curcumin to TPGS (1:10, *w*/*w*). All formulations shown in Figure 2B resulted in a similar solubility of curcumin, regardless of the mannitol content, indicating that mannitol did not readily affect the solubility of curcumin in solid dispersion formulations. However, because solid dispersion (SD) I, SD II, and SD III (Figure 2C) had a pasty appearance, it seemed that these could cause problems in the handling and development of a solid dosage form. In contrast, using a higher amount of mannitol (SD IV and SD V, Figure 2C) gave the formulation powderier characteristics. A composition of curcumin, TPGS, and mannitol, at a ratio of 1:10:15 (*w*/*w*/*w*), was chosen as the final formulation for further investigation.

### 2.2. Characterizations of Curcumin-Loaded Solid Dispersion Formulation

#### 2.2.1. Differential Scanning Calorimetry (DSC)

**Figure 3:**
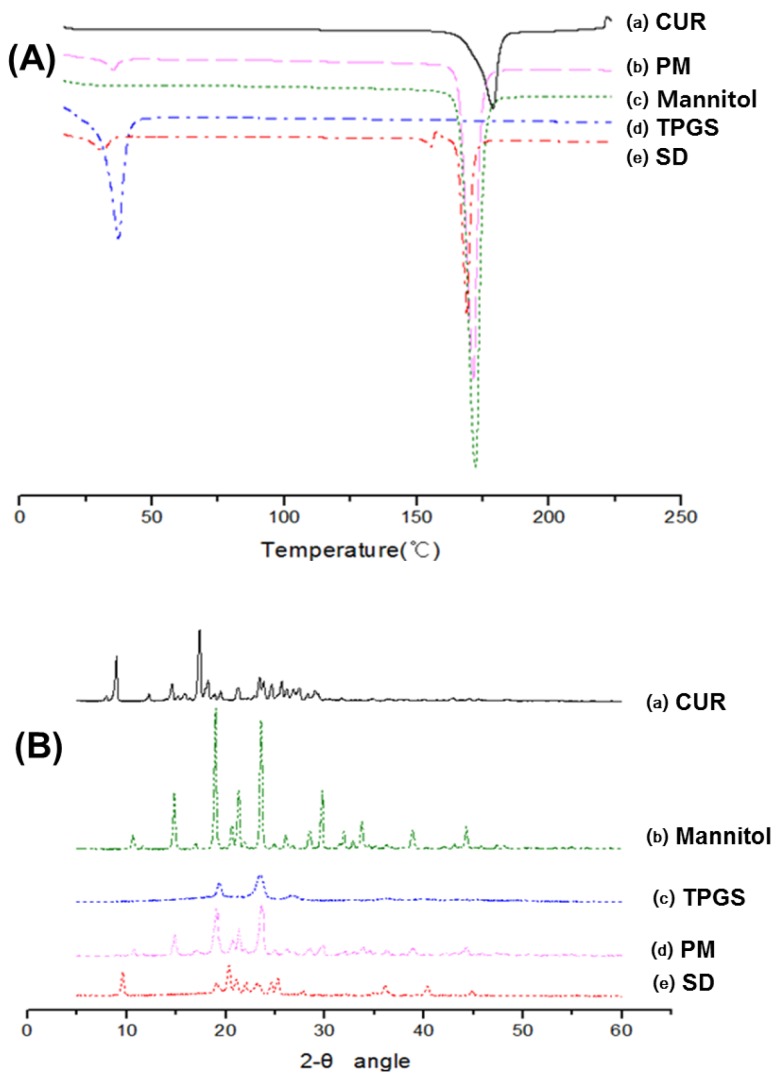
(**A**) Differential scanning calorimetry (DSC) thermograms of: (a) curcumin; (b) physical mixture (PM) of curcumin:TPGS:mannitol = 1:10:15 (*w*/*w*/*w*); (c) mannitol; (d) TPGS; and (e) solid dispersion (SD) of curcumin:TPGS:mannitol = 1:10:15 (*w*/*w*/*w*); (**B**) X-ray diffraction (XRD) patterns of: (a) curcumin; (b) mannitol; (c) TPGS; (d) physical mixture of curcumin:TPGS:mannitol = 1:10:15 (*w*/*w*/*w*); and (e) solid dispersion of curcumin:TPGS:mannitol = 1:10:15 (*w*/*w*/*w*).

The DSC curve featured a sharp endothermic peak at around 178 °C, 170 °C and 39 °C corresponding to melting point of curcumin, mannitol and TPGS, respectively, indicating their crystalline nature (Figure 3A-(a,c,d)) [1,23,24,25,26]. The DSC thermograms of the physical mixture (Figure 3A-b) and solid dispersion (Figure 3A-e) displayed similar patterns, i.e., disappearance of the endothermic peak of curcumin and reduced endothermic peak of mannitol and TPGS. These results might be attributed to curcumin dissolving in the molten TPGS and mannitol during DSC measurement [20,27].

#### 2.2.2. X-ray Diffraction (XRD)

The X-ray diffraction patterns of curcumin, mannitol, TPGS, physical mixture, and solid dispersion are shown in Figure 3B. Pure curcumin exhibited sharp peaks in a 2Θ range of 5–30, indicating a typical crystalline structure (Figure 3B-a) [28]. Additionally, TPGS and mannitol were present in the crystalline form (Figure 3B-b,c) [25,29]. The various diffraction peaks observed in the individual XRD spectrum were also observed (at lower intensity) in that of the physical mixture, suggesting an absence of chemical interaction among curcumin, TPGS, and mannitol (Figure 3B-d). The diffraction peaks of the solid dispersion decreased markedly compared with those of the physical mixture, implying that all ingredients in the solid dispersion were in the reduced crystalline state (Figure 3B-e).

#### 2.2.3. pH Dependence of Curcumin Solubility

**Figure 4:**
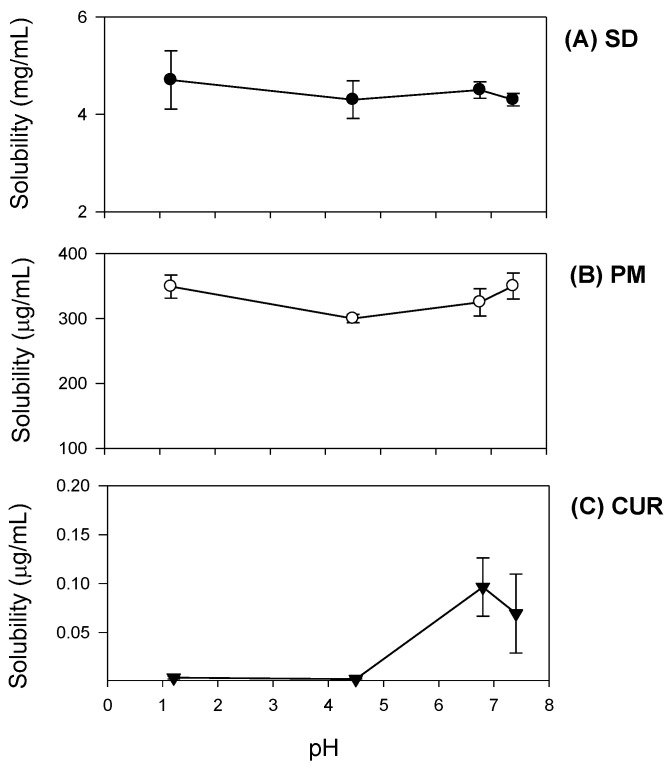
pH-dependent solubility of curcumin was determined for curcumin (CUR, ▼; **A**); physical mixture of curcumin:TPGS:mannitol = 1:10:15 (*w*/*w*/*w*) (PM, ○; **B**); and curcumin-loaded solid dispersion (SD, ●; **C**) in the pH range of 1.2–7.4. Each data point represents the mean ± S.D. of three independent experiments.

The solubility of curcumin remained unchanged when measured at pH 1.2 and 4.5. However, it increased 25.1-fold at pH 6.8, similar to its solubility at pH 7.4 (Figure 4C). The solubility of the curcumin-loaded solid dispersion markedly increased across a pH range 1.2–7.4, and pH-dependent solubility was not observed, in contrast to the solubility of curcumin itself (Figure 4A). In the physical mixture, the curcumin pH solubility pattern was similar to that in the solid dispersion, while its solubility was 10-fold lower than that of curcumin in the solid dispersion (Figure 4B). These results suggested that the presence of hydrophilic excipients such as TPGS and mannitol markedly increased curcumin solubility and diminished the pH-dependent solubility pattern. Thus, the limited and pH dependent solubility of pure curcumin could be greatly improved by formulating it as a solid dispersion.

#### 2.2.4. Dissolution Studies

**Figure 5:**
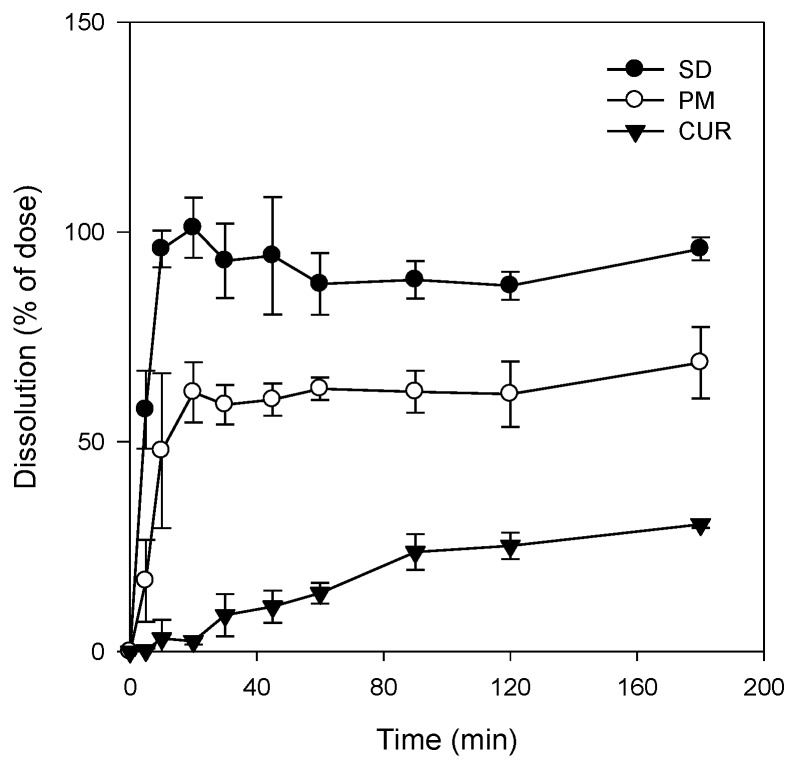
Dissolution profile of curcumin was determined for curcumin (CUR, ▼), physical mixture of curcumin:TPGS:mannitol = 1:10:15 (PM, ○), and solid dispersion formulation (SD, ●). Each data point represents the mean ± S.D. of three independent experiments.

We observed the dissolution profiles of curcumin, the curcumin solid dispersion formulation, and the corresponding physical mixture, which are shown in Figure 5. The dissolved fraction of pure curcumin remained at <30% for 180 min. The dissolution rate of curcumin in the physical mixture was higher than that of pure curcumin, due to the presence of TPGS and mannitol. However, the solid dispersion showed a significantly greater dissolution rate compared with those of curcumin powder and the physical mixture. The increase in dissolution rate of the solid dispersion versus the corresponding physical mixture was attributable to changes in the crystalline state, as evidenced in Figure 3.

### 2.3. Pharmacokinetic Studies

**Figure 6:**
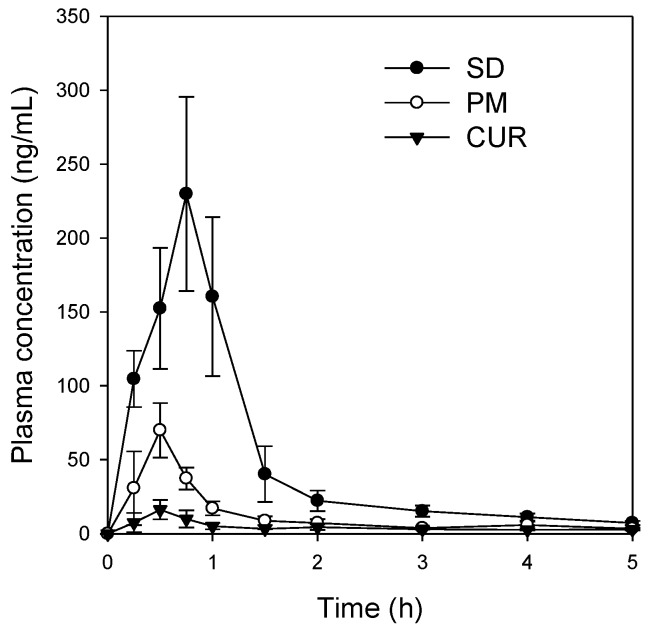
Plasma concentration-time profile of curcumin after oral administration of curcumin (CUR, ▼), physical mixture of curcumin:TPGS:mannitol = 1:10:15 (PM, ○), and solid dispersion formulation (SD, ●). Curcumin suspended in 1% carboxymethyl cellulose was administered orally to rats at a dose of 200 mg/kg. Solid dispersion formulation and corresponding physical mixture (at a ratio of curcumin:TPGS:mannitol = 1:10:15) was administered orally to rats at a dose of 30 mg/kg. Each data point represents the mean ± S.D. of three to four rats.

The plasma concentration profiles of curcumin powder, the physical mixture, and the solid dispersions are shown in Figure 6. The solid dispersions exhibited improved pharmacokinetic behavior over both curcumin powder and the physical mixture; the pharmacokinetic parameters are summarized in Table 1. About 26- and 86-fold increases in C_max_/dose were reported for the physical mixture and solid dispersion formulation compared to the values for the curcumin powder, respectively. The AUC_∞_/dose for the physical mixture and solid dispersion formulation increased by approximately 22- and 65-fold compared to the value reported for the curcumin powder, respectively, with no significant change in time to reach C_max_ (T_max_) or half-life. These results suggest that the increases in C_max_/dose and AUC_∞_/dose for the curcumin-loaded solid dispersion formulation were likely due to increased absorption of curcumin because of the increased solubility and dissolution of the solid dispersion formulation.

### 2.4. Enhanced Permeability of Curcumin-Loaded Solid Dispersion

**Figure 7:**
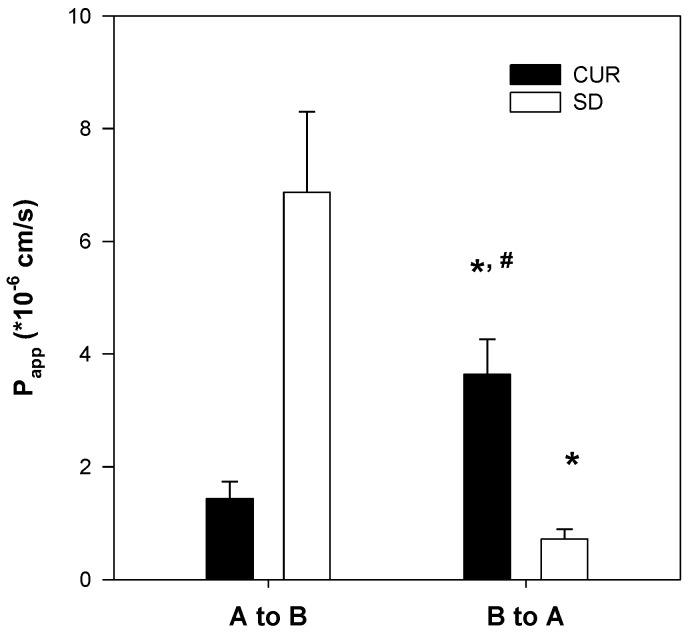
Effect of solid dispersion formulation on the permeability of curcumin in Caco-2 cell monolayers. A to B and B to A permeability (P_app_) of curcumin alone (CUR) and solid dispersion formulation (SD) (20 μM curcumin each) were measured in Caco-2 cells. Each data point represents the mean ± S.D. of three independent experiments. * *p* < 0.01, statistically significant compared with curcumin group. ^#^ *p* < 0.01, statistically significant compared with P_app, AB_.

The apparent permeability (P_app_) of basal to apical (B to A) secretion of curcumin was 2.5-fold greater than the apical to basal (A to B) permeability (1.4 × 10^−6^ cm/s; Figure 7). These results suggest that the absorption of curcumin could be restricted by the involvement of an efflux system, such as P-gp, consistent with previous reports [13,14]. The A to B P_app_ of curcumin determined for the curcumin-loaded solid dispersion was 4.8-fold greater than that of curcumin itself. Moreover, the B to A P_app_ of the curcumin-loaded solid dispersion decreased by 90% (Figure 7). These results indicated that the intestinal absorption of curcumin could be increased through inhibition of the efflux pump by the solid dispersion formulation.

### 2.5. Modulation of P-gp by Curcumin-Loaded Solid Dispersion

**Figure 8:**
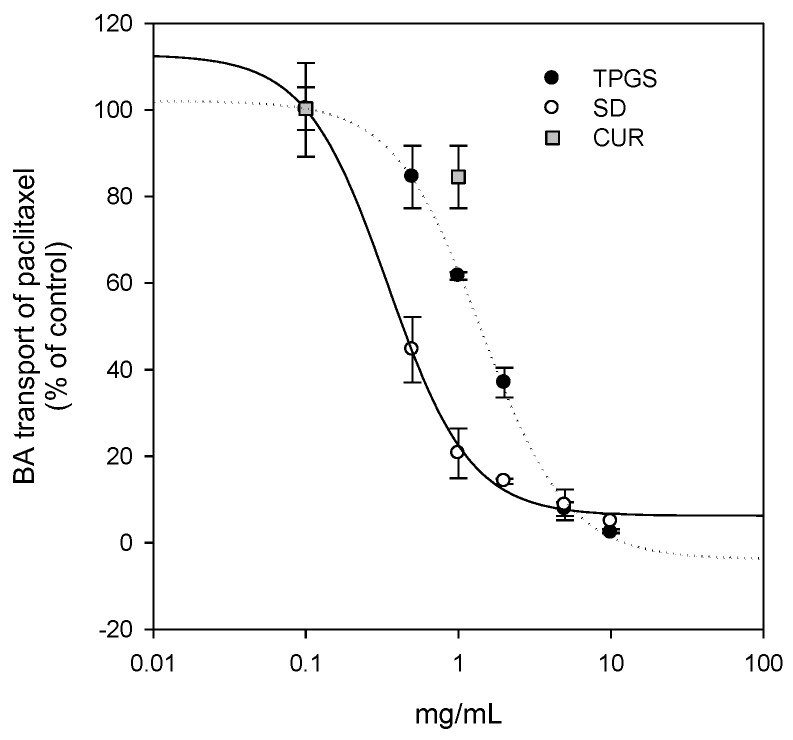
Inhibitory effect of TPGS (●) and solid dispersion formulation (SD, ○) on P-gp transport activity. B to A transport rate of 5 μM paclitaxel was measured in the presence of curcumin (0.1 and 1 mg/mL), TPGS (0.1, 0.5, 1, 2, 5, and 10 mg/mL), or solid dispersion formulation (0.1, 0.5, 1, 2, 5, and 10 mg/mL as TPGS) in LLC-PK1-P-gp cells. Each data point represents the mean ± S.D. of three independent experiments.

To investigate the P-gp modulation by the solid dispersion formulation, P-gp-mediated paclitaxel efflux (i.e., B to A transport rate of paclitaxel in this study) in P-gp overexpressed LLC-PK1-P-gp cells was measured in the presence of curcumin, TPGS, and its solid dispersion formulation [30]. As shown in Figure 8, curcumin, TPGS, and the solid dispersion formulation inhibited P-gp-mediated B to A transport of paclitaxel in a dose-dependent manner. Comparison of the inhibitory potencies of 1 mg/mL curcumin and TPGS showed that they inhibited paclitaxel transport by 16.4% and 38.4%, respectively. However, the presence of solid dispersion formulation inhibited paclitaxel transport by 79.3%. The IC_50_ values for the inhibition of B to A transport of paclitaxel were calculated as 1.4 mg/mL for TPGS and 0.35 mg/mL for the solid dispersion formulation, suggesting the enhancement of P-gp inhibitory potency by the solid dispersion formulation. The IC_50_ value of curcumin could not be calculated because of its solubility problem.

**Figure 9:**
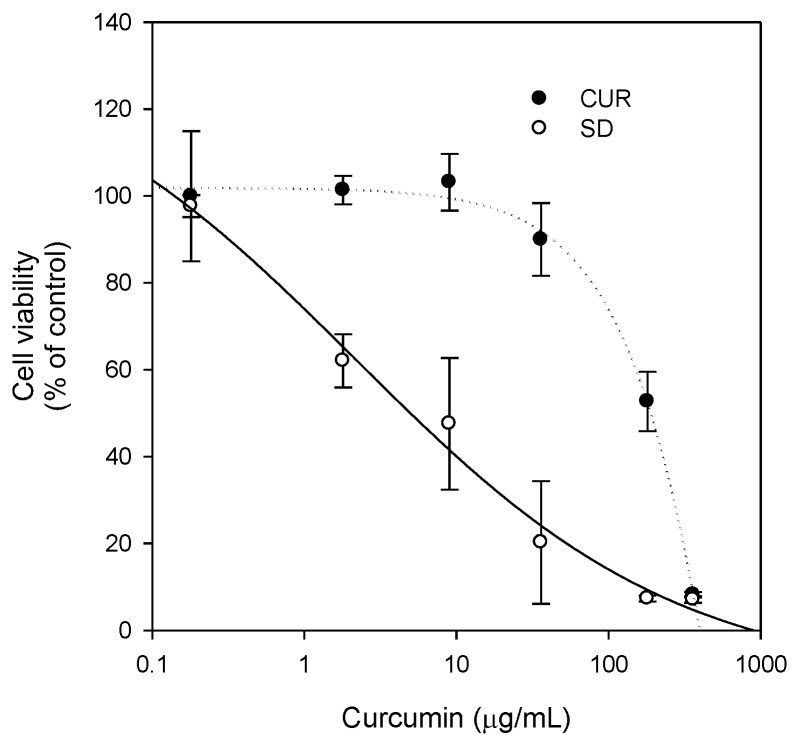
The effect of curcumin (CUR, ●) or solid dispersion formulation (SD, ○) on viability of MDA-MB-231 cells. MTT assay was performed after 24 h incubation in the presence of curcumin (0.18, 1.8, 9, 36, 180 and 360 μg/mL), 10-fold amount of TPGS, and their solid dispersion formulation. Each data point represents the mean ± S.D. of three independent experiments.

Next, we investigated whether the solid dispersion formulation increased the anti-proliferative effect of curcumin in MDA-MB-231 breast cancer cells. Curcumin has been reported to be effective against breast cancer [6], and the overexpression of P-gp in MDA-MB-231 cells is related to anti-cancer drug resistance [31,32]. The cytotoxic effect of curcumin was enhanced by treating as a curcumin-loaded solid dispersion formulation. The results were also consistent with a decreased IC_50_ value of curcumin-loaded solid dispersion (5.2 μg/mL) compared with the value reported for curcumin itself (185 μg/mL) (Figure 9).

**Figure 10:**
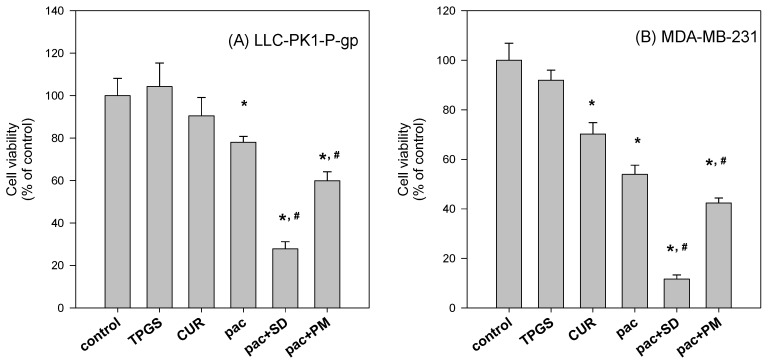
The differential anti-proliferative effect of solid dispersion formulation from physical mixture in LLC-PK1-P-gp (**A**) and MDA-MB-231 cells (**B**). LLC-PK1-P-gp and MDA-MB-231 cells were grown in 96 well plates (10^4^ cells/well) and incubated with paclitaxel (pac; 500 nM) in the absence and presence of solid dispersion (SD; curcumin:TPGS:mannitol = 0.09:0.9:1.35 mg/mL), and physical mixture (PM) for 2 h. For comparison, curcumin (CUR; 0.09 mg/mL) and TPGS (0.9 mg/mL) were also incubated for 2 h. After the 2 h incubation, the medium was replaced with fresh medium and incubated for 22 h. MTT assay was performed after 24 h incubation. * *p* < 0.01, statistically significant compared with control group. ^#^ *p* < 0.01, statistically significant compared with paclitaxel treatment.

Additionally, the differential effect between curcumin-loaded solid dispersion and the physical mixture was investigated. We hypothesized that the intracellular concentrations of curcumin and paclitaxel would vary in short-term dosing with paclitaxel and either curcumin powder, curcumin-loaded solid dispersion, or their physical mixture since the dissolution rate and solubility of curcumin were different for the three preparations. LLC-PK1-P-gp and MDA-MB-231 cells were incubated with paclitaxel and curcumin formulation for 2 h, followed by the replacement of incubation medium with fresh medium and further incubated for 22 h [33] The cytotoxic effect of intracellular paclitaxel and curcumin (which were accumulated for 2 h) was measured by cell viability. As the results demonstrate, the cytotoxic effect was greater for paclitaxel co-incubated with curcumin-loaded solid dispersion than for co-incubation with the physical mixture or curcumin and TPGS itself (Figure 10). The results suggested that the cellular content of paclitaxel in the presence of curcumin-loaded solid dispersion was higher than that in the presence of the physical mixture. The increased solubility and dissolution rate of curcumin-loaded solid dispersion formulation caused greater P-gp inhibition, facilitating intracellular paclitaxel accumulation and enhancing paclitaxel’s cytotoxic effect.

## 3. Discussion

The strategy of enhancing aqueous solubility and cell permeability is the most reasonable way to overcome low oral bioavailability and enhance the therapeutic efficacy of phytochemical drugs. According to the literature, increasing the solubility and modulation of P-gp-mediated efflux are important for improving the oral bioavailability and efficacy of curcumin [7,8,9,10]. Therefore, the purpose of this study was to develop a solid dispersion formulation of curcumin using FDA-approved safe excipients with the ability to enhance curcumin solubility and to inhibit P-gp function. TPGS and mannitol were selected for this purpose as a surfactant and P-gp modulator, and as a hydrophilic carrier, respectively.

Since it is advantageous in solid dispersion systems to achieve a molecular level dispersion of the drug in the carrier matrix, the drug and carrier must be homogeneously mixed during the preparation of the solid dispersion [34]. Because no suitable solvent is available for dissolving curcumin, TPGS, and mannitol together, curcumin-loaded solid dispersion was formulated by a two-step procedure, consisting of solvent evaporation and freeze-drying. Curcumin and TPGS were first dissolved in acetone, and a mixture of curcumin and TPGS was obtained through solvent evaporation. This mixture and mannitol were then dissolved in water. Finally, the curcumin-loaded solid dispersion formulation was prepared from a clear homogeneous solution of curcumin, TPGS, and mannitol through a freeze-drying method at a weight ratio of 1:10:15. Previously, Seo et al. reported that the solubility of curcumin could be increased using a surfactant with an HLB value in the range of 13–16 [20]. The HLB value of TPGS is 13.2 [19], well within the above range.

Additionally, curcumin exhibited pH-dependent solubility; specifically, the solubility of curcumin increased in a pH-dependent manner. This suggests that orally administered curcumin may become solubilized after passing through the stomach, which would result in delayed absorption. However, the presence of TPGS and mannitol markedly increased curcumin solubility and diminished its pH-dependency (Figure 4). As a result, the dissolution profile of curcumin (Figure 5) in its pure form was quite different from that reported for the solid dispersion. The maximum release of curcumin was ~90% within 10 min for the solid dispersion and <30% over 80 min for pure curcumin. The results were consistent with the increases in C_max_/D and AUC_∞_/D observed for the solid dispersion compared with those for curcumin alone (Table 1). Increased intestinal permeability also contributed to enhanced curcumin bioavailability in the solid dispersion compared with that reported for curcumin itself. Pharmaceutical excipients, such as TPGS, are known to increase the absorption of curcumin by modulating the efflux of this compound from Caco-2 cells [16,17]. Thus, the inhibition of P-gp-mediated curcumin efflux by TPGS (Figure 7) could also contribute to the increase in curcumin permeability. Taken together, the increased plasma concentration and bioavailability of curcumin could be explained by increases in the solubility, dissolution rate, and permeability of curcumin in the solid dispersion formulation with TPGS.

Additionally, since curcumin was known to be effective against breast cancer, we compared the therapeutic efficacy of curcumin-loaded solid dispersion with that of curcumin itself. As shown in Figure 8, the inhibition curve derived from the solid dispersion formulation was shifted to the left compared with that of curcumin and TPGS, suggesting that the curcumin-loaded solid dispersion has enhanced P-gp inhibitory effects compared with that reported for both curcumin and TPGS. Higher TPGS content may have contributed to the inhibition of P-gp-mediated B to A transport of paclitaxel. Consequently, the intracellular content of curcumin increased (0.81 ± 0.09 ng/10^6^ cells for curcumin only vs. 2.72 ± 0.59 ng/10^6^ cells for curcumin solid dispersion formulation in LLC-PK1-P-gp cells), which could explain the increased therapeutic effect of the solid dispersion formulation. This result was consistent with the increased anti-proliferative effect of curcumin-loaded solid dispersion formulation in MDA-MB-231 breast cancer cells compared with that of curcumin only (Figure 9).

To further investigate the beneficial effect of the solid dispersion formulation compared with that of a physical mixture, an MTT assay with a two-step incubation of the solid dispersion and physical mixture was performed. The dissolution rate of curcumin in curcumin powder, physical mixture, and solid dispersion formulation differed markedly. Therefore, this varying dissolution would result in differential cellular uptake of curcumin and paclitaxel for 2 h. The variable cellular uptake of these drugs depending on formulation status might cause differences in cell viability, as demonstrated in Figure 10.

In conclusion, a solid dispersion preparation of curcumin with TPGS and mannitol could be an effective way of overcoming the limitations of curcumin alone such as low solubility, dissolution, and oral absorption. Moreover, increased cellular accumulation of curcumin and the P-gp inhibitory effect of this solid dispersion formulation may further potentiate the curcumin response and other anti-cancer drugs such as paclitaxel.

## 4. Materials and Methods

### 4.1. Materials

Curcumin, TPGS, d-mannitol (98%), and paclitaxel were obtained from Sigma-Aldrich Chemical Co. (St. Louis, MO, USA). Dimethyl sulfoxide (DMSO), 3-(4,5-dimethylthiazol-2-yl)-2,5-diphenyltetrazolium bromide (MTT), and Hank’s balanced salt solution were also purchased from Sigma-Aldrich. Acetone was obtained from Merck KGaA (Darmstadt, Germany). Acetonitrile, methanol, and pure water were obtained from Fisher Scientific Korea, Ltd. (Seoul, Korea) and were of high-performance liquid chromatography (HPLC) grade. All other chemicals used were of analytical grade.

Caco-2 cells were purchased from American Type Culture Collection (Rockville, MD, USA). LLC-PK1-P-gp cells were purchased from Corning (Corning, NY, USA) and MDA-MB-231 cells were obtained from the Korean Cell Line Bank (Seoul, Korea). Fetal bovine serum and Dulbecco’s Modified Eagle’s medium (DMEM), Medium 199, penicillin–streptomycin, and Trypsin–EDTA were purchased from Hyclone Laboratories (Logan, UT, USA).

### 4.2. Preparation of Solid Dispersions

#### 4.2.1. Solubilizing Effect of TPGS on Curcumin

To confirm the solubilizing effect of TPGS on curcumin, the solubility of curcumin was investigated in aqueous solutions of TPGS. The concentrations of TPGS in aqueous solution ranged 0.005% to 5% (*w*/*v*). Excess amounts of curcumin (about 10 mg) were added to 5 mL of TPGS aqueous solution, shaken in an air bath at 25 °C for 5 h, centrifuged at 13,200 rpm for 10 min (6415R, Eppendorf, Hamburg, Germany) and filtered through a nylon membrane filter (0.45 μm). The filtrates were suitably diluted with acetonitrile into the calibration curve range, and analyzed for curcumin with LC-MS/MS, as described below. Additionally, excess amounts of curcumin and TPGS mixture (equivalent to 20 mg as curcumin, Figure 2A) were added to 3 mL of water, shaken in an air bath at 25 °C for 5 h, centrifuged at 13,200 rpm for 10 min (6415R) and filtered through a nylon membrane filter (0.45 μm). These filtrates were also diluted with acetonitrile into the calibration curve range, and analyzed for curcumin with LC-MS/MS.

#### 4.2.2. Preparation of Curcumin-Loaded Solid Dispersions

Curcumin-loaded solid dispersions were formulated in a two-step procedure, consisting of solvent evaporation and freeze-drying. Initially, curcumin and TPGS were accurately weighed (0.5 g and 5 g, respectively), and dissolved in acetone. The solution was evaporated using a rotary evaporator (N-1110V-W, EYELA, Tokyo, Japan). Subsequently, 7.5 g of mannitol and distilled water was added to the dried waxy residue and stirred to obtain a clear solution. The solution was frozen at −80 °C for 6 h in a deep freezer (MDF-U50V, Sanyo, Osaka, Japan) and freeze-dried at −120 °C for 72 h using a chemical-free freeze-dryer (FDCF-12012, Operon, Gyeonggi-do, Korea). After freeze-drying, the resulting samples were passed through a KP sieve (mesh size = 0.84 μm) and stored in a desiccator. For comparison, a physical mixture of curcumin, TPGS, and mannitol in the same ratio as the solid dispersion formulation was prepared by accurate weighing and thorough kneading. The physical mixture was sieved through a KP sieve (mesh size = 0.84 μm) and stored in a desiccator.

### 4.3. Characterization of Curcumin-Loaded Solid Dispersion

#### 4.3.1. Solubility Studies

Excess amounts of curcumin-loaded solid dispersions equivalent to 20 mg as curcumin (Figure 2B) were added to 3 mL of water. They were shaken in an air bath at 25 °C for 5 h, centrifuged at 13,200 rpm for 10 min and filtered through a nylon membrane filter (0.45 μm). The filtrates were suitably diluted with acetonitrile into the calibration curve range, and analyzed for curcumin with LC-MS/MS, as described below.

#### 4.3.2. Differential Scanning Calorimetry (DSC)

Differential scanning calorimetry (DSC) measurements were performed using a DSC 131EVO (Setaram, Caluire, France). Sample weighing approximately 5 mg were placed in a closed aluminum pan and heated at a scanning rate of 3 °C/min from 25 °C to 225 °C, with nitrogen purging at 20 mL/min. Indium was used to calibrate the temperature scale.

#### 4.3.3. X-ray Diffraction (XRD)

X-ray diffraction (XRD) was performed at room temperature with an X-ray diffractometer (Ultima IV; Rigaku Co., Tokyo, Japan) using Cu Kα radiation, generated at 40 mA and 40 kV. Data were obtained from 5° to 60° (2θ) at a step size of 0.02° and a scanning speed of 5°/min.

#### 4.3.4. Dissolution Studies

The dissolution studies were performed using a United States Pharmacopeia (USP) dissolution paddle method. Briefly, dissolution studies were carried out in 900 mL distilled water containing 0.01% Tween 80 for 180 min in a D-63150 dissolution test apparatus (Erweka, Heusenstamm, Germany) at 37 °C. Samples were tested in three groups; curcumin powder, solid dispersion formulation, and the physical mixture, respectively. Varying amounts of each preparation, each equivalent to 20 mg curcumin, were packed into a gelatin capsule and each capsule was then placed inside the sinker. Samples were collected at 5, 10, 20, 30, 40, 60, 90, 120, and 180 min. An aliquot (5 mL) of the medium was collected and filtered through a nylon membrane filter (0.45 μm). The filtrates were suitably diluted with acetonitrile to the calibration curve range, and analyzed for curcumin content with LC-MS/MS. An equal volume of fresh medium was replaced after each sampling.

### 4.4. Pharmacokinetics Studies

Sprague Dawley rats (males, 8 weeks old, 250–280 g; Orient Bio, Seoul, Korea) were acclimatized for 1 week in an animal facility at the College of Pharmacy, Kyungpook National University. All procedures involving animals were approved by the Animal Care and Use Committee of the Kyungpook National University (No. 2014-0043-1, approved on 22 May 2014).

The rats were fasted for at least 12 h before the oral administration of drugs and were allowed water ad libitum. The femoral vein and artery were cannulated using polyethylene tubes (PE-50; Jungdo, Seoul, Korea) under light isoflurane anesthesia, and heparinized saline (10 U/mL) was used to prevent blood clotting.

Curcumin powder was suspended in 1% carboxymethyl-cellulose (CMC) solution and was administered to rats at a dose of 200 mg/kg via oral gavage. The curcumin-loaded solid dispersion and a physical mixture were suspended in water and were administered at a dose of 30 mg/kg as curcumin. Blood samples were collected from the femoral artery at 0, 0.25, 0.5, 0.75, 1, 1.5, 2, 3, 4, and 5 h following oral administration and centrifuged at 13,000 rpm for 5 min to prepare plasma samples. Plasma samples (50 μL) were collected and stored at −80 °C until analysis. After thawed in water bath, aliquots of plasma (50 μL) were added to 250 μL of acetonitrile containing 0.2 ng/mL of propranolol (internal standard) and the mixture was vortexed for 10 min. After centrifugation at 13,200 rpm for 10 min, an aliquot of the sample (2 μL) was injected directly into the LC-MS/MS system.

### 4.5. Permeability Studies

Caco-2 cells were grown in tissue culture flasks in DMEM, supplemented with 20% fetal bovine serum, 1% penicillin–streptomycin, 4 mM l-glutamine, and 1% non-essential amino acids. After reaching 70% confluency, cells were seeded at a density of 5 × 10^5^ cells/insert onto the membrane inserts of 12-Transwell plates. Culture medium was changed every 2 days for 21 days. The integrity of the cell monolayers was evaluated by measuring transepithelial electrical resistance and those with values in the range of 300–650 Ω·cm^2^ were used in the transport experiments [35].

Aliquots (0.5 mL) of Hank’s balanced salt solution (HBSS) containing curcumin alone or a curcumin-loaded solid dispersion (equivalent to 20 μM curcumin) were added to the insert and 1.5 mL of HBSS without curcumin was added to the basal side. The apical to basal (A to B) transport of curcumin was measured by transferring the insert to the next well containing fresh HBSS medium every 15 min for 1 h. Aliquots (1.5 mL) of HBSS containing curcumin or its formulation (equivalent to 20 μM curcumin) were added to the basal side, and 0.5 mL of HBSS without curcumin was added to the insert side. The basal to apical (B to A) transport was measured by sampling 0.4 mL of HBSS medium on the apical side and it was replaced with 0.4 mL of fresh HBSS every 15 min for 1 h. Aliquots (100 μL) of each sample were added to a 100 μL acetonitrile solution containing 2 ng/mL propranolol (internal standard). After vortex mixing and centrifugation, the supernatant was injected directly into the LC-MS/MS system.

### 4.6. P-gp Inhibition by Solid Dispersion Formulation

To investigate the effect of curcumin formulation on the P-gp transport activity, we measured the basal to apical (B to A) transport of paclitaxel in LLC-PK1-P-gp cell monolayers. Aliquots (1.5 mL) of HBSS medium containing 5 μM paclitaxel in the presence of TPGS (0.1, 0.5, 1, 2, 5, or 10 mg/mL) or curcumin-TPGS solid dispersion formulation (0.1, 0.5, 1, 2, 5, 10 mg/mL TPGS) were added to the basal side, and 0.5 mL of fresh HBSS medium was added to the apical side. The transport medium in the apical side was replaced with 0.35 mL of fresh incubation medium every 15 min for 1 h. Aliquots (100 μL) of samples were added to a 100 μL acetonitrile containing 2 ng/mL of propranolol (internal standard). After vortex-mixing for 10 min and centrifugation for 10 min at 13,000 rpm, the supernatant was injected directly into the LC-MS/MS system for the quantification of paclitaxel.

### 4.7. Analysis of Curcumin and Paclitaxel Using LC-MS/MS

Curcumin concentrations were analyzed using an Agilent 6430 Triple Quadrupole LC-MS/MS system (Agilent, Wilmington, DE, USA) equipped with an Agilent 1260 HPLC system. Separation was performed on a Hydro-RP column (2.0 mm × 100 mm, 2.5 μm; Phenomenex, Torrance, CA, USA) using a mobile phase consisting of water and acetonitrile (40:60 *v*/*v*) with 0.1% formic acid at a flow rate of 0.25 mL/min. Quantification was carried out using multiple reaction monitoring (MRM) at *m*/*z* 369.2 → 285.1 for curcumin and *m*/*z* 260.0 → 116.0 for propranolol in positive ionization mode. The lower limit of quantification (LLOQ) was determined to be 5 ng/mL and the standard curve exhibited linearity over the range of 5–1000 ng/mL. Intra- and inter-day precision and accuracy had coefficients of variance of less than 15%.

Paclitaxel concentrations were analyzed using an Agilent 6430 Triple Quadrupole LC-MS/MS system. Separation was performed in a Polar RP column (2.0 mm × 150 mm, 5 μm, Phenomenex) using a mobile phase that consisted of water and methanol (10:90, *v*/*v*) with 0.1% formic acid at a flow rate of 0.2 mL/min. Mass spectra were recorded by electrospray ionization in positive mode. Quantification was carried out using MRM mode at *m*/*z* 876.4 → 308.1 for paclitaxel and *m*/*z* 260.0 → 116.0 for propranolol. In this study, the LLOQ was determined to be 5 ng/mL and the standard curve exhibited linearity over the range 5–2000 ng/mL. Intra- and inter-day precision and accuracy had coefficients of variance of less than 15%.

### 4.8. Cell Viability Test

MDA-MB-231 cells, grown in 96-well plates (10^4^ cells/well), were incubated in the presence of curcumin (0.18, 1.8, 9, 36, 180, and 360 μg/mL), 10-fold quantities of TPGS, and solid dispersion formulation for 24 h. After the 24 h incubation, the medium was replaced with 200 μL MTT (0.5 mg/mL) and the plate was incubated for 4 h. The medium was removed and the purple formazan products were solubilized with 120 μL DMSO. Cell contents were measured by the absorbance at 570 nm. Cell viability was indicated as percentage of live cells in the sample compared with controls after background correction.

Additional testing was performed to compare the P-gp inhibitory effects of solid dispersion formulations and physical mixtures. To this end, we assessed the anti-proliferative effect of paclitaxel in MDA-MB-231 and LLC-PK1-P-gp cells, grown in 96-well plates (10^4^ cells/well), and incubated with paclitaxel (500 nM) in the absence and presence of solid dispersion (curcumin:TPGS:mannitol = 0.09:0.9:1.35 mg/mL), and its physical mixture for 2 h. To document the unwanted cytotoxic effect of excipients, curcumin (0.09 mg/mL) and TPGS (0.9 mg/mL) were also incubated for 2 h. After the 2 h incubation, the medium was replaced with fresh medium and the sample was further incubated for 22 h. The final process was identical to the above-described method involving the addition of MTT.

### 4.9. Data Analysis

Pharmacokinetic parameters were determined using a non-compartmental analysis (WinNonlin 2.0; Pharsight, Mountain View, CA, USA). The area under the plasma concentration-time curve, from zero to the last sampling time (AUC_last_), was calculated using the linear trapezoidal method, and AUC from last time to infinity (AUC_last−∞_) was estimated by dividing the last measured concentration in plasma by the terminal rate constant. The terminal elimination half-life (t_½_) was calculated from the slope of the terminal phase. The maximum plasma concentration (C_max_) and time to reach C_max_ (T_max_) were read directly from the experimental data [30].

The apparent permeability (P_app_) of the drug was calculated by dividing the initial drug transport rate (V, pmol/cm^2^·min) by the initial drug concentration in the donor compartment of the insert (C) multiplied by the surface area of the insert (A): $P_{\mathrm{app}}=\frac{V}{C\times A}$ [30].

All data are expressed as means ± standard deviation (SD) of three independent experiments. Differences between treatments were evaluated using the unpaired *t*-test. A *p* value < 0.05 was considered to indicate statistical significance.

## Figures and Tables

**Table 1:** Pharmacokinetic parameters of curcumin following per oral administration of curcumin, curcumin-loaded solid dispersion and its physical mixture.

Treatment	Curcumin	Physical Mixture	Curcumin-Loaded Solid Dispersion
Dose (mg/kg)	200	30	30
C_max_ (ng/mL)	17.9 ± 5.3	69.9 ± 18.4	233 ± 62.4
C_max_/D (ng/mL/mg/kg)	0.09 ± 0.03	2.33 ± 0.61 *	7.77 ± 2.08 *
T_max_ (h)	0.44 ± 0.13	0.50 ± 0.00	0.63 ± 0.14
t_1/2_ (h)	1.61 ± 0.74	3.66 ± 2.16	1.93 ± 0.33
AUC_last_ (ng·h/mL)	19.9 ± 4.77	60.5 ± 17.3	240 ± 30.2
AUC_∞_ (ng·h/mL)	26.8 ± 6.84	89.0 ± 43.3	262 ± 35.3
AUC_∞_/D (ng·h/mL/mg/kg)	0.13 ± 0.03	2.97 ± 1.44 *	8.73 ± 1.18 *

Data are presented as mean ± S.D., *n* = 3–4; C_max_, maximum plasma concentration; T_max_, time to reach C_max_; AUC, area under the plasma concentration–time curve; C_max_/D and AUC_∞_/D, dose normalized C_max_ and AUC_∞_, respectively; t_1/2_, terminal elimination half-life. * *p* < 0.05 compared with the curcumin group.
